# Epidemiology, classification and treatment of patella fractures: an observational study of 3194 fractures from the Swedish Fracture Register

**DOI:** 10.1007/s00068-022-01993-0

**Published:** 2022-05-30

**Authors:** Mark Kruse, Olof Wolf, Sebastian Mukka, Anders Brüggemann

**Affiliations:** 1 Department of Surgical and Perioperative Sciences at Umeå University, Umeå, Sweden; 2 Section of Orthopaedics, Department of Surgical Sciences, Uppsala University, Uppsala, Sweden

**Keywords:** Patella fractures, Fracture epidemiology, Trauma mechanism, Fracture treatment, Swedish Fracture Register, Knee fracture

## Abstract

**Background:**

Basic epidemiological data on patella fractures derived from large nationwide and multicenter studies are scarce. This observational register study describes patient epidemiology, fracture classification and treatment of patella fractures in adults in a Swedish population.

**Methods:**

We conducted an observational study on data derived from the Swedish Fracture Register (SFR) on all patella fractures classified as non-periprosthetic and non-pathological, registered between 2014 and 2018 in individuals aged ≥ 18years. Epidemiological data on sex, age, side, seasonal variation, trauma mechanism, fracture classification (according to AO/OTA), and treatment were analyzed.

**Results:**

3194 patella fractures were analyzed, occurring at a median age of 67 (range 19–100) years. 64% of all patients were female. Most fractures were caused by low-energy trauma, with 70% due to falling from a standing height. 1796 (56%) of the fractures were transverse compared to 845 (26%) vertical fractures. Most fractures (*N*=2148, 67%) were treated non-operatively. Operative treatment consisted mainly of Tension Band Wiring (TBW) performed in 774 (24%) patients.

**Conclusions:**

Patella fractures mainly occur in elderly women (> 65 years), commonly caused by low-energy trauma. The main treatment is non-operative (67%), except for transverse (AO/OTA C3) fractures. TBW remains the most used operative treatment of choice. These results may help health care providers, researchers and clinicians better understand the panorama of patella fractures in Sweden.

**Supplementary Information:**

The online version contains supplementary material available at 10.1007/s00068-022-01993-0.

## Introduction

The patella is a vital part of the biomechanical excursion of the extensor apparatus of the knee [1]. A fractured patella can disrupt the extensor mechanism in combination with incongruent posterior articular surface and thus cause long-term complications, e.g., discomfort due to femoropatellar osteoarthritis [2, 3]. The established treatment options depend on the underlying fracture pattern. They include non-operative treatment for vertical and undisplaced fractures, whereas operative treatment is mainly reserved for displaced fractures with a disrupted extensor mechanism [4]. Operative treatment methods range from sutures and tension band wiring (TBW) to recently popularized plate fixation [5]. Several case series report a high degree of secondary surgery, mainly implant removal due to prominent hardware [6, 7]. There is also an increased risk of posttraumatic osteoarthritis and later need of total knee arthroplasty (TKA) [3]. There are few large studies on the epidemiology of patella fractures [8, 9].

This study describes the injury mechanism, fracture classification, sex and age distribution, seasonal variation and primary treatment in patients with patella fracture using data derived from the Swedish Fracture Register (SFR).

## Materials and methods

### Swedish fracture register

This observational register study was based on data derived from the SFR, which was established in 2011. The SFR holds information on injury mechanism, fracture classification and treatment on an individual level based on the unique Swedish personal identity number (PIN). Several studies have found the registration in the SFR to have high accuracy and validity [10–12]. The coverage was 50% of orthopedic departments at the start of the study in 2014 and > 80% at the end of the study in 2018. More than 330,000 fractures had been registered by the end of 2018. Information on peri-implant and periprosthetic fractures as well as open fractures according to the Gustilo–Anderson classification is available.

### Patient selection

**Fig. 1:**
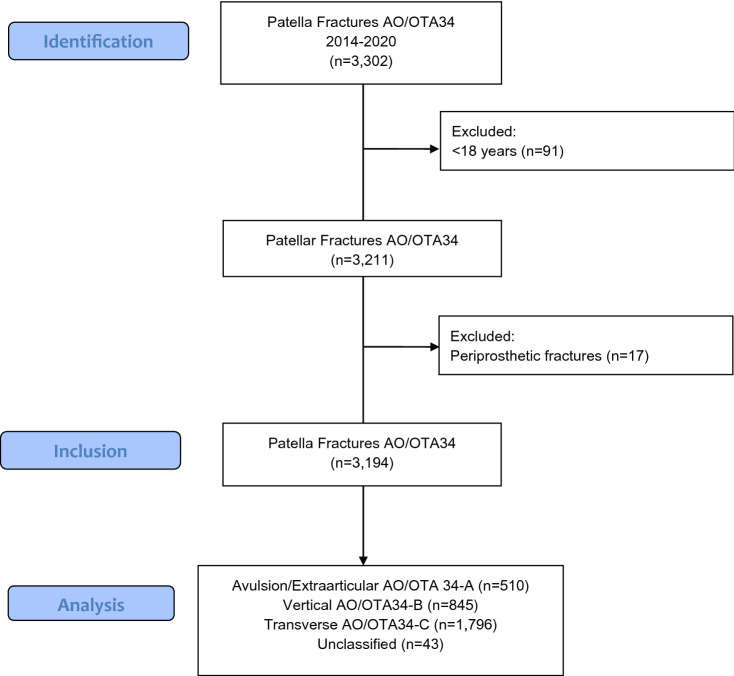
Flow diagram of patient selection process

All open and closed patellar fractures (ICD S82.00-01, AO/OTA 34A-C) registered in the SFR as sustained from January 1st, 2014 to December 31st, 2018 in patients aged ≥ 18 years were included. We excluded all periprosthetic fractures (Fig. 1).

### Study variables

The following variables were retrieved from the SFR: age, sex, date of injury, trauma mechanism, fracture classification including side, open/closed fractures and treatment. The injury mechanism was categorized as a simple fall, an unspecified fall, road traffic accident, or other cause. Classification of fractures according to AO [13] included extra-articular fragment/avulsion (AO/OTA 34A1-2), vertical (lateral, medial; AO/OTA 34B1-2) and transverse (simple, multifragmentary, wedge; AO/OTA 34C1-3). Open fractures were classified according to Gustilo–Anderson [14].

Treatment was divided into non-operative or operative treatment. Operative treatment methods are further registered as TBW, screw fixation, plate fixation and combined fixation method. Non-operative treatment was not further specified.

### Statistics

Nominal variables are presented as proportions of all fractures and scale variables as medians with ranges. We used the R version 4.0.3 (R Core Team, Vienna, Austria) software package for statistical analyses.

### Ethics

The study was performed according to the updated version of the Helsinki declaration. Approval was given by the Swedish Ethical Review Authority (dnr:2015/509 and 2019/01106).

## Results

### Study subjects and descriptive data

**Fig. 2:**
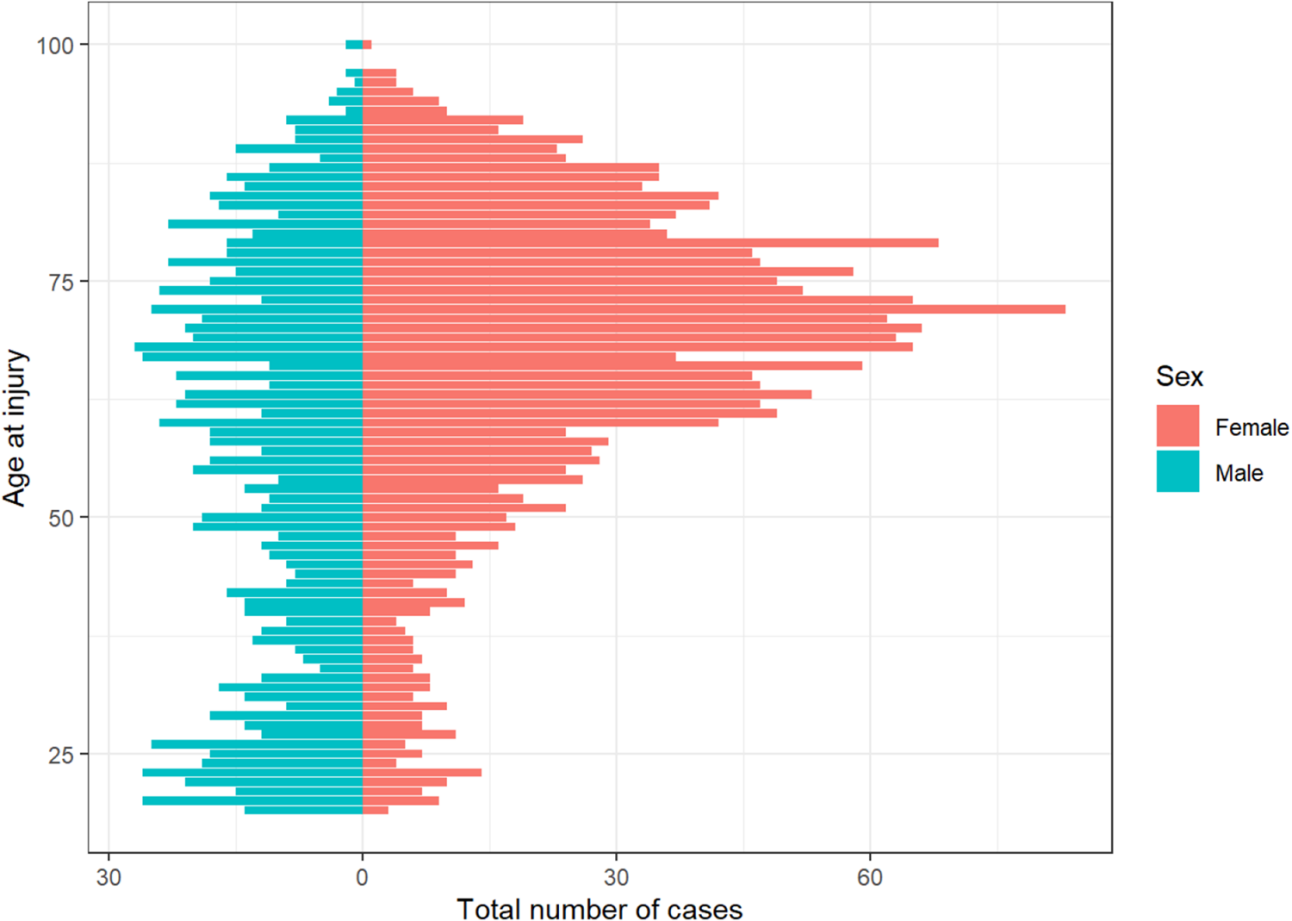
Age distribution at injury

3302 patella fractures were extracted from the SFR (ICD-10 S82.00-01). After excluding peri-prosthetic fractures, the final sample comprised 3194 patients (Fig. 1). Median age was 67 (range 19–100) years. 64% of the patients were women with a median age of 70 (19–100) years and 36% were males with a median age of 59 (19–100) years (Table 1). There was a peak in the elderly (> 60 years) population for women with patella fractures compared to the slightly biconvex curve in men (Fig. 2). 70% of the fractures were caused by a simple fall. This proportion was higher in women: 75% of the women sustained their fracture due to a simple fall compared to 60% in men. In contrast, road traffic accidents (RTA) caused 4% of fractures in women and 13% in men (Table 2) and 11% of the multifragmentary C3 fractures were caused by an RTA compared to 5% of the horizontal simple C1 fractures (Table 3). 85% of the fractures were a result of low energy trauma (Table 1). Only about 1 in 20 fractures was registered as sustained by high energy trauma.

**Table 1:** Patient epidemiology and trauma mechanism

	Female (*N* = 2039)	Male (*N* = 1155)	Overall (*N* = 3194)
Age at injury			
Median [min–max]	70.0 [19.0–100]	59.0 [19.0–100]	67.0 [19.0–100]
Side			
Left	1068 (52.4%)	578 (50.0%)	1646 (51.5%)
Right	971 (47.6%)	577 (50.0%)	1548 (48.5%)
Mechanism			
Fall from height	167 (8.2%)	112 (9.7%)	279 (8.7%)
Fall standing height	1530 (75.0%)	700 (60.6%)	2230 (69.8%)
Road traffic accident	85 (4.2%)	155 (13.4%)	240 (7.5%)
Other cause	105 (5.1%)	143 (12.4%)	248 (7.8%)
Stress fracture	7 (0.3%)	9 (0.8%)	16 (0.5%)
Unspecified fall	172 (8.4%)	89 (7.7%)	261 (8.2%)
Type			
High energy	50 (2.5%)	130 (11.3%)	180 (5.6%)
Low energy	1825 (89.5%)	902 (78.1%)	2727 (85.4%)
Unknown	164 (8.0%)	123 (10.6%)	287 (9.0%)

**Table 2:** AO/OTA classification

	Female (*N* = 2039)	Male (*N* = 1155)	Overall (*N* = 3194)
A1	268 (13.1%)	137 (11.9%)	405 (12.7%)
A2	56 (2.7%)	49 (4.2%)	105 (3.3%)
B1	349 (17.1%)	279 (24.2%)	628 (19.7%)
B2	132 (6.5%)	85 (7.4%)	217 (6.8%)
C1	682 (33.4%)	261 (22.6%)	943 (29.5%)
C2	234 (11.5%)	119 (10.3%)	353 (11.1%)
C3	296 (14.5%)	204 (17.7%)	500 (15.7%)
Unclassified	22 (1.1%)	21 (1.8%)	43 (1.3%)

**Table 3:** AO/OTA classification according to trauma mechanism

	Avulsion	Extraarticular fragment	Vertical lateral	Vertical medial	Horizontal simple	Horizontal wedge	Horizontal multifragmentary	Not classified	Overall
	(*N* = 405)	(*N* = 105)	(*N* = 628)	(*N* = 217)	(*N* = 943)	(*N* = 353)	(*N* = 500)	(*N* = 43)	(*N* = 3194)
Cause									
Fall same level	269 (66.4%)	69 (65.7%)	428 (68.2%)	150 (69.1%)	692 (73.4%)	262 (74.2%)	339 (67.8%)	21 (48.8%)	2230 (69.8%)
Fall from height	44 (10.9%)	8 (7.6%)	52 (8.3%)	14 (6.5%)	81 (8.6%)	29 (8.2%)	49 (9.8%)	2 (4.7%)	279 (8.7%)
Unspecified Fall	28 (6.9%)	8 (7.6%)	71 (11.3%)	10 (4.6%)	72 (7.6%)	27 (7.6%)	37 (7.4%)	8 (18.6%)	261 (8.2%)
Road traffic accident (RTA)	28 (6.9%)	9 (8.7%)	44 (7.2%)	26 (12.1%)	50 (5.3%)	22 (6.2%)	54 (10.8%)	3 (6.9%)	236 (7.3%)
Exposure to external forces	21 (5.1%)	8 (7.7%)	18 (2.8%)	12 (5.5%)	24 (2.5%)	8 (2.3%)	15 (3.0%)	8 (18.7%)	114 (3.6%)
Assault, legal intervention, operation of war	1 (0.2%)	0 (0%)	1 (0.2%)	1 (0.5%)	2 (0.2%)	0 (0%)	1 (0.2%)	0 (0%)	6 (0.2%)
Missing	14 (3.5%)	3 (2.9%)	14 (2.2%)	4 (1.8%)	22 (2.3%)	5 (1.4%)	5 (1.0%)	1 (2.3%)	68 (2.1%)

The injury was most often sustained in the patient’s home (29%), followed by public streets and other places (e.g., farming industries, government buildings and hospitals). For 32% of the fractures, the injury location was unspecified.

### Seasonal variation

**Fig. 3:**
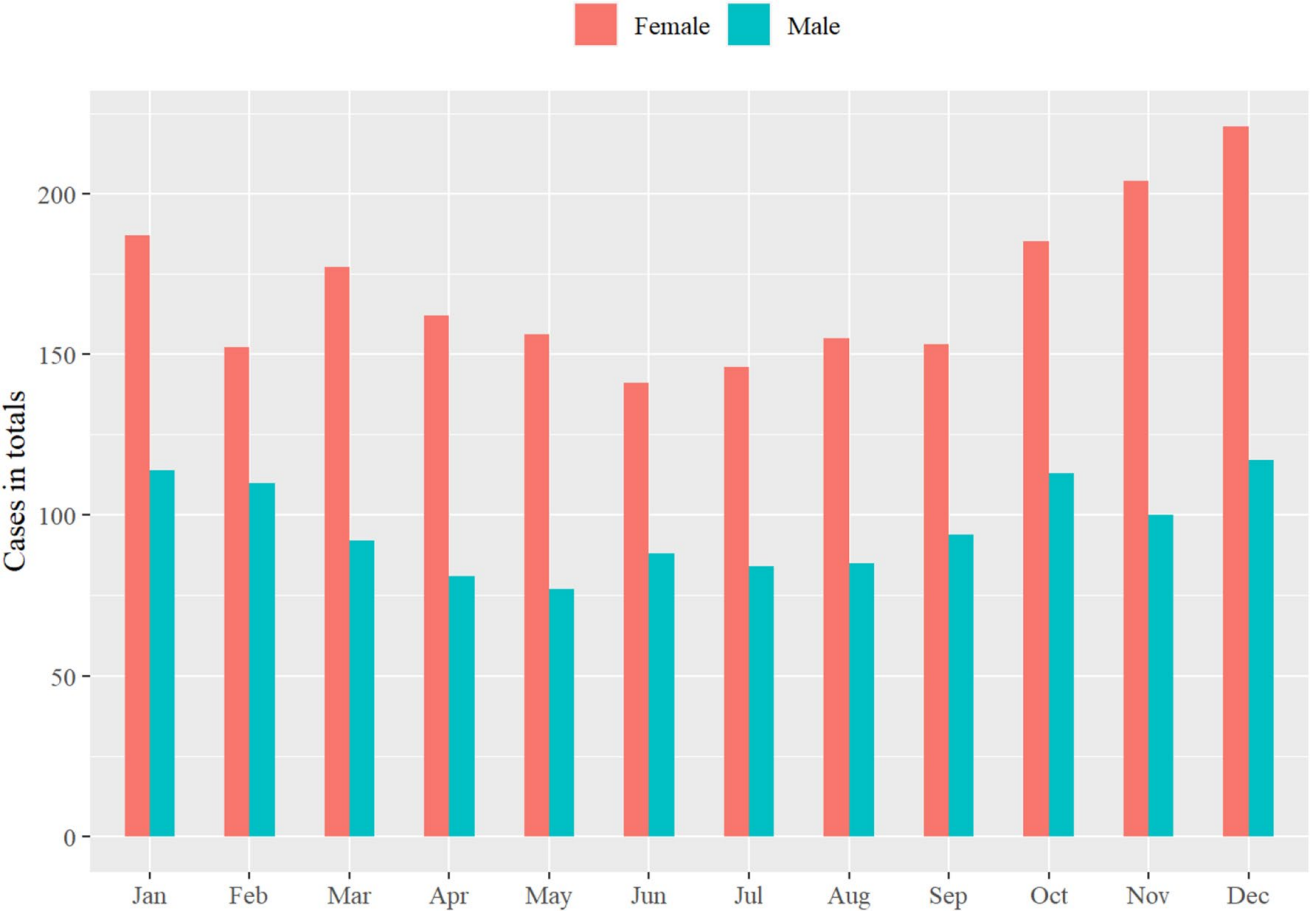
Seasonal variation of patella fractures

More fractures occurred in the colder months (October–March), which was more pronounced in women (Fig. 3).

### Fracture classification

16% of the patella fractures were A-type fractures, of which the majority were avulsion fractures (13%, Table 2). The vertical B-type fractures accounted for 27% of all fractures, most located laterally (20%). The lateral vertical fractures occurred in 24% of the fractures in men compared to only 17% in women. More than half of all fractures were transverse C-type fractures (56%), with 30% simple transverse fractures. Women sustained more simple transverse fractures than men (33% versus 23%). The more comminuted C3 fractures were found in 16% of all patella fractures.

### Treatment

Two thirds of all patella fractures were treated non-operatively. Almost all A- and B-type fractures were treated non-operatively, except for 1 of 5 inferior pole avulsion fractures treated with fixation (Table 4). For C-type fractures, half of the fractures were treated non-operatively. Operative treatment increased with increasing fracture complexity, with TBW being the preferred method in most cases (C1—35%; C2—43%; C3—45%). Plate fixation was rare and almost exclusively used in C3-type fractures. Combined fixation methods were used in up to 5% of the fractures in C2-type fractures.

**Table 4:** Treatment of patella fractures according to AO/OTA classification

	34-A1 (*N* = 405) 12.7%	34-A2 (*N* = 105) 3.3%	34-B1 (*N* = 628) 19.7%	34-B2 (*N* = 217) 6.8%	34-C1 (*N* = 943) 29.5%	34-C2 (*N* = 353) 11.1%	34-C3 (*N* = 500) 15.6%	Unclassified (*N* = 43) 1.3%	Overall (*N* = 3194)
Treatment									
Non operative	309 (76.3%)	89 (84.8%)	610 (97.1%)	196 (90.3%)	534 (56.6%)	164 (46.5%)	207 (41.4%)	29 (67.4%)	2138 (66.9%)
Tension band wiring	59 (14.6%)	2 (1.9%)	2 (0.3%)	2 (0.9%)	326 (34.6%)	150 (42.5%)	227 (45.4%)	2 (4.7%)	770 (24.1%)
Screw fixation	3 (0.7%)	0 (0%)	3 (0.5%)	3 (1.4%)	9 (1.0%)	2 (0.6%)	0 (0%)	1 (2.3%)	21 (0.7%)
Plate fixation	1 (0.2%)	0 (0%)	2 (0.3%)	0 (0%)	3 (0.3%)	1 (0.3%)	16 (3.2%)	0 (0%)	23 (0.7%)
Combined fixation	16 (4.0%)	1 (1.0%)	2 (0.3%)	1 (0.5%)	24 (2.5%)	16 (4.5%)	21 (4.2%)	0 (0%)	81 (2.5%)
Missing	17 (4.2%)	13 (12.4%)	9 (1.4%)	15 (6.9%)	47 (5.0%)	20 (5.7%)	29 (5.8%)	11 (25.6%)	161 (5.0%)

## Discussion

### Epidemiology

This register-based observational study confirms that the patella fracture mainly occurs in older women (> 65 years) due to low-energy trauma caused by a simple fall. Outdoor slips could cause seasonal variation and main trauma mechanism with low-energy fall in the winter months. Many fractures with potential intact extensor mechanisms are treated non-operatively. When surgery was chosen, patients were mostly treated with TBW.

Higher frequencies of patella fractures in females have been reported in concordance with our finding [8, 15]. For instance, Begnér et al. reported a 141% increase of patella fractures in females between the 1950s and 1980s [15]. Other studies on operatively treated patella fractures present a more even sex distribution [16, 17]. This difference could be interpreted as a difference in fracture classification between sexes with more severe or displaced fractures in males which would thus be considered more suitable for operative intervention. We found a sex difference with more vertical lateral fractures occurring in men and more simple transverse fractures occurring in women. Larsen et al. also found a biconvex curve of patella fracture incidence over age in men, consistent with our findings [8]. Several authors have argued that patella fractures, especially in females, should be considered an osteoporotic fracture because of the female population’s higher age and frequency of low energy trauma [8, 15, 16]. Patella fractures in children and adolescents could be viewed as a different entity caused by other trauma mechanisms in patients with a different physiological setting and a different sex distribution [18].

Only 1 in 20 patella fractures was caused by high energy, most of which were multi-fragmentary horizontal fractures. Our finding that the most common injury mechanism was a simple fall is directly in line with previous studies [8]. Road traffic accidents as a cause for patella fractures were three times more common in males. Again, the sex difference in fracture distribution may in part be due to different bone quality: a simple fall and direct blow resulting in a lateral vertical fracture (men) or a simple transverse fracture (women).

### Fracture classification

Classifying patella fractures is challenging, evidenced by the low intraobserver reproducibility, where adding CT scans can alter both classification and treatment selection [19]. Transverse fracture patterns (34-C) represented just over half of the fractures in our study compared to just over 60% of the fractures in a Danish study [8]. That Danish study reported 25% comminuted C3 fractures and 14% high energy injuries, which are higher proportions than our study, explaining the difference in complex fracture distribution.

### Treatment

Half of the fractures with a potential disruption of the extensor mechanism (C-type) were treated non-operatively compared to almost all fractures without extensor mechanism affection (A2 and B-type fractures). Of the horizontal fractures, articular congruency and non-displaced fractures are assumed to be present in those treated non-operatively [4]. Vestergaard et al. [9] reported 74% non-operatively treated patellar fractures, which agrees with our findings.

TBW as the golden standard in operative treatment is widely debated, mainly because of the high frequency of reoperations due to implant removal [20]. The use of cannulated screws in TBW seems favorable to Kirschner wires (K-wires) as the former minimize local irritation and thus the need for subsequent implant extraction [7, 21]. Combining cannulated screws with sutures (i.e., Fiberwire, Arthrex, Naples, FL, USA) instead of metal banding could reduce complication rates even further without increasing the risk of malunion [22]. However, the technique is technically challenging and has specific pitfalls: the length of screws and wear on sutures must be considered [7]. More comminuted fractures are not amenable for screw fixation. A combination of sutures with K-wires could, therefore, be a valid option [23]. A shift in the applied methods from TBW [16] to plating has been observed by several authors [5, 24–26] but was not found in our study. A randomized controlled or register-based randomized controlled study with adequate sample size is needed to compare the outcomes of different operative techniques for osteosynthesis of patella fractures. Zhan et al. [27] described three-dimensional CT mapping for the multi-fragmentary patellar fractures pre-operatively aiding in the choice of surgical treatment and using the rarely involved supero-medial corner of the patella as a cornerstone for fixation.

Larsen et al. [28] did not find any increased mortality in the elderly. Still, the patella fracture can cause long-term discomfort with lower health-related quality of life [2] and increased risk for knee arthroplasty compared to patients without patella fractures [3]. This underlines the importance of adequate initial treatment.

### Strengths and limitations

Our study presents with several shortcomings. Regrettably, we do neither have data on complications and reoperations in this data set nor on other variables, such as length of stay or procedural costs or—most notably—patient satisfaction in terms av PROMs. One major limitation is the fact that the SFR does not comment on associated ligament injuries. This in its turn introduces uncertainty regarding the treatment applied which could very well be influenced by the aforementioned associated injuries. Miscoding, underreporting and transferring errors are examples for sources of error inherent to all observational studies and our study is no exemption. Finally, the data set has missing data (5% or 161 fractures) on treatment, but with 3194 fractures with injury mechanism and fracture classification, we still find the overall epidemiological description of value for the involved stakeholders, i.e., patients, health care providers and orthopedic surgeons in concordance with Aitken et al. [29]. This observational study on national register data is the largest epidemiological study on patella fractures reported. The study period ranges from timepoints in which the SFR had a coverage of 50% to over 80% of the orthopedics departments and thereby gave a broad national description of the patella fracture population and treatment.

## Conclusions

Patella fractures are mainly caused by a simple low energy fall in elderly woman. Most patients are treated non-operatively. In the case of operative treatment, TBW is the most common method. These results may help health care providers, researchers and clinicians to better understand the panorama of patella fractures in Sweden.

## Supplementary Information

Below is the link to the electronic supplementary material.

Supplementary file1 (PDF 20 kb)
