# Supplementary material for: Epidemiology, classification and treatment of patella fractures: an observational study of 3194 fractures from the Swedish Fracture Register
Source: Eur J Trauma Emerg Surg. 2022 May 30;48(6):4727–34. doi: 10.1007/s00068-022-01993-0 (PMC9712342; doi:10.1007/s00068-022-01993-0)
Supplement: Supplementary file 1 — Supplementary file1 (PDF 20 kb) [file 68_2022_1993_MOESM1_ESM.pdf]

Supplementary Table 1 - Fracture pattern and Surgical Procedure

	**Avulsion**<br><br>**(N=405)**	**Extraarticular fragment**<br><br>**(N=105)**	**Vertical lateral**<br><br>**(N=628)**
**Procedure**			
Cast, brace, other bandaging	309 (76.3%)	89 (84.8%)	610 (97.2%)
TBW	59 (14.6%)	2 (1.9%)	2 (0.3%)
Combined method	4 (1.0%)	0 (0%)	1 (0.2%)
Other surgery	12 (3.0%)	1 (1.0%)	1 (0.2%)
Plate fixation	1 (0.2%)	0 (0%)	2 (0.3%)
Screw fixation	3 (0.7%)	0 (0%)	3 (0.5%)
Excision surgery	5 (1.2%)	3 (2.9%)	0 (0%)
Fixation cartilage	0 (0%)	1 (1.0%)	1 (0.2%)
Synovial surgery, artroscopy	1 (0.2%)	0 (0%)	0 (0%)
External fixation	0 (0%)	0 (0%)	0 (0%)
Missing	11 (2.7%)	9 (8.6%)	8 (1.3%)

Vertical medial (N=217)	Horizontal simple (N=943)	Horizontal wedge (N=353)	Horizontal multifragmentary (N=500)	Not classified (N=43)	Overall (N=3194)
196 (90.4%)	534 (56.6%)	164 (46.4%)	207 (41.4%)	29 (67.4%)	2138 (66.9%)
2 (0.9%)	326 (34.6%)	150 (42.5%)	227 (45.4%)	2 (4.7%)	770 (24.1%)
0 (0%)	20 (2.1%)	15 (4.2%)	13 (2.6%)	0 (0%)	53 (1.7%)
1 (0.5%)	4 (0.4%)	1 (0.3%)	8 (1.6%)	0 (0%)	28 (0.8%)
0 (0%)	3 (0.3%)	1 (0.3%)	16 (3.2%)	0 (0%)	23 (0.7%)
3 (1.4%)	9 (1.0%)	2 (0.6%)	0 (0%)	1 (2.3%)	21 (0.7%)
3 (1.4%)	0 (0%)	0 (0%)	2 (0.4%)	0 (0%)	13 (0.4%)
3 (1.4%)	2 (0.2%)	0 (0%)	1 (0.2%)	1 (2.3%)	9 (0.3%)
1 (0.5%)	0 (0%)	0 (0%)	0 (0%)	1 (2.3%)	3 (0.1%)
0 (0%)	1 (0.1%)	0 (0%)	0 (0%)	0 (0%)	1 (0.0%)
8 (3.7%)	44 (4.7%)	20 (5.7%)	26 (5.2%)	9 (20.9%)	135 (4.2%)
